# Empagliflozin does not reverse lipotoxicity-induced impairment in human myeloid angiogenic cell bioenergetics

**DOI:** 10.1186/s12933-022-01461-4

**Published:** 2022-02-17

**Authors:** Gloria Cinquegrani, Valentina Spigoni, Federica Fantuzzi, Riccardo C. Bonadonna, Alessandra Dei Cas

**Affiliations:** 1 Endocrinology and Metabolism, Department of Medicine and Surgery, University of Parma, Via Gramsci 14, 43126 Parma, Italy; 2 Division of Endocrinology and Metabolic Diseases, Azienda Ospedaliero-Universitaria of Parma, Via Gramsci 14, 43126 Parma, Italy

**Keywords:** Myeloid angiogenic cells, Empagliflozin, Stearic acid, Cell metabolism, Oxygen consumption

## Abstract

**Background:**

Empagliflozin can curb inflammation and oxidative stress, through sodium-proton exchanger (NHE) inhibition, in a model of lipotoxicity in human myeloid angiogenic cells (MAC), which mediate endothelial repairing processes. Aim of this study is to assess in human MAC whether: (1) Stearic acid (SA) induced inflammation and increase in oxidant stress is accompanied by bioenergetic alterations; (2) empagliflozin anti-lipotoxic action is concomitant with coherent changes in bioenergetic metabolism, possibly via NHE blockade.

**Methods:**

MAC were isolated from peripheral blood of healthy volunteers and incubated in the presence/absence of SA (100 μM for 3 h) with/without empagliflozin (EMPA 100 μM) or amiloride (Ami 100 μM) for 1 h. Cell respiration (oxygen consumption rate OCR) and anaerobic glycolysis (measured as proton production rate) were recorded in real-time by Seahorse technology, and ATP production (anaerobic glycolysis- and oxphos-derived) rates were calculated.

**Results:**

SA, at the concentration causing inflammation and increased oxidant stress, altered cell bioenergetics of human MAC, with overall reductions in basal OCR and oxphos-derived ATP production (all p < 0.05), pointing to mitochondrial alterations. EMPA, at the concentration counteracting SA-induced lipotoxicity, both alone and in the presence of SA, caused NHE-independent extensive bioenergetic alterations (from p < 0.05 to p < 0.01), greater than those induced by SA alone.

**Conclusions:**

In human MAC: (1) SA altered cell bioenergetics, concomitantly with inflammation and oxidant stress; (2) EMPA possibly inhibited mitochondrial respiration, (3) the protective effect of EMPA against SA-induced lipotoxicity was unlikely to be mediated through bioenergetic metabolism.

## Introduction

Immunometabolism—defined as the interplay between immunological and metabolic processes—aims at identifying the precise mechanisms by which cell-intrinsic metabolic processes and the function of immune cells influence each other [1]. Particularly, a growing number of findings highlights the crucial role of metabolic reprogramming in monocyte-derived cell (e.g. macrophages) function showing a close interconnection between metabolic pathways and inflammation (*metaflammation*) in both innate and adaptive immune responses [2]. Myeloid angiogenic cells (MAC) are monocyte-originated circulating cells which play a key role in the maintenance of vascular homeostasis in response to different stress stimuli, mainly through paracrine mechanisms (i.e. secretion of pro-angiogenic factors and cytokines) [3]. Importantly, not only an impaired number and function of MAC is associated and predict future cardiovascular (CV) events [4–6], but dysfunctional MAC also release pro-inflammatory mediators leading to atherogenic inflammation and plaque complication [7]. In an experimental model of MAC lipotoxicity [8]—a condition characterized by elevated free fatty acid levels underlying the association among insulin resistance, endothelial dysfunction and CV risk [9]—we reported that physiological concentrations of stearic acid (SA: 18:0) induced inflammation, oxidant stress and apoptosis and were associated with impaired cell function. The plausible association between this lipotoxic dysfunctional pro-inflammatory response and changes in MAC bioenergetics was not investigated.

Sodium-glucose cotransporter-2 inhibitors (SGLT2-I) are glucose-lowering agents, which act increasing urinary glucose excretion by inhibiting renal glucose reabsorption. In several placebo-controlled CV outcomes trials [10–12], conducted at glucose equipoise in patients with type 2 diabetes mellitus (T2D) and proved atherosclerotic cardiovascular disease, most SGLT2-I, but one, reduced major adverse CV events, suggesting a class effect [13]. Among the possible mechanisms of action underlying SGLT2-I CV benefits, some pre-clinical evidence reports direct, glucose-independent, anti-inflammatory and anti-oxidative stress effects. Dapagliflozin decreased LPS-stimulated expression of IL-1β and NOD-like receptor-3 (Nlrp3 inflammasome) via AMP-activated protein kinase (AMPK) activation in mouse cardiac fibroblasts [14], and, both empagliflozin and dapagliflozin, at clinically relevant concentrations, reduced reactive oxygen species (ROS) generation in TNFα-stimulated human coronary arterial endothelial cells [15]. Of note, reported SGLT2-I effects on cell metabolism are discordant [16–18].

We recently demonstrated that SGLT2-I (empagliflozin and dapagliflozin) exert a strong anti-inflammatory and anti-oxidative action in a model of SA-induced lipotoxic MAC. These effects are SGLT2 independent and possibly involve one or more isoforms of the sodium-proton exchangers (NHE), as a molecular transducer [19]. Based on these premises we hypothesized that: a. SA-induced lipotoxicity might alter MAC bioenergetic phenotype, and b. antagonism of SA-induced lipotoxicity by SGLT2-I might be concomitant with coherent changes in MAC metabolism.

To pursue these aims, we assessed several key bioenergetic fluxes [20, 21] in MAC with/without exposure to SA and in presence/absence of SGLT2-I.

## Materials and methods

### Cell isolation and culture

MAC were isolated and cultured according to published methods [3, 5], as previously described [8, 22–24]. Briefly, mononuclear cells (MNCs) were isolated by Lymphoprep (Euroclone, Milano, Italy) density gradient centrifugation from healthy donors’ buffy-coats and grown into fibronectin-coated dishes at a density of 1 × 10^7^ cells/well. MNCs were grown in endothelial cell growth medium-2 (EGM-2) with supplements (Lonza, Milano, Italy) at 37 °C in a humidified 5% CO_2_ incubator for 7 days. On day 7, MAC appeared in culture as adherent cells displaying an elongated spindle-shaped morphology.

### Metabolic assessment

#### Cell preparation

At day 7, 24 h prior to assay, MAC were detached with Accutase solution (Euroclone) and re-seeded in fibronectin-coated Seahorse Flux microplates (Agilent, Santa Clara, CA, USA) at a density of 6 × 10^4^ cells/microwell. One hour prior to assays, cells were washed and incubated in a final volume of 180 μl of XF DMEM Basal medium (seahorse specific, Agilent), after pH adjustment at 7.4. Glucose and glutamine (both 5.5 mM) and pyruvate (0.37 mM) were added to the DMEM assay medium to mirror the respective concentrations in the EGM-2 growth medium.

#### Culture conditions

MAC were pre-treated with/without empagliflozin (EMPA, Selleck Chemicals, Houston, TX, USA) 100 μM or amiloride (Ami; Cayman Chemical Company, Ann Arbor, MI, USA) 100 μM for 1 h, in the presence/absence of stearic acid (SA) 100 µM for further 3 h. The concentration of 100 µM of EMPA was selected on the base of preliminary experiments [19] in which a concentration (1–10–100 µM)-response study showed that only EMPA 100 µM strongly reduced inflammation in stearate-treated MAC. Moreover, the viability assays revealed no cytotoxic effect of the tested concentrations of EMPA on MAC [19]. Vehicle (DMSO 0.1%)-treated cells were used as control.

#### Stearate solution preparation

Stearate stock solution was prepared by dissolving SA (Sigma-Aldrich, St Louis, MO, USA) in 0.1 M NaOH at 73 °C for 30 min. Then, SA 5 mM was complexed to 10% fatty acid-free bovine serum albumin (BSA) as previously reported [8, 25].

#### Cell Mito Stress Test

In order to investigate mitochondrial function in the different culture conditions (control, SA, Ami, EMPA, EMPA + SA, Ami+SA), a Cell Mito Stress test (Seahorse XFp Analyzer, Agilent), was carried out following manufacturer's instructions [26]. Assay readouts—oxygen consumption rate (OCR) and extracellular acidification rate (ECAR)—were measured in MAC after serial addition of 2 μM FCCP (an uncoupling agent which disrupts the mitochondrial transmembrane potential and stimulates the maximal respiration) and 1 μM rotenone/antimycin A, which inhibit complex I and complex III of the electron transport chain (ETC). All OCR and ECAR data were normalized to 6 × 10^4^ cells (viability assay).

### Viability assay

A viability assay was performed to normalize the number of viable cells in each microwell at the end of the Mitostress test. The VisionBlue TM Fluorescence Cell Viability Assay Kit (BioVision, Mountain View, CA) was performed following manufacturer's instructions, as already reported [22, 23]. Briefly, microwell medium was discarded and replaced with 100 μl of fresh culture medium (EGM-2) plus 10 μl of VisionBlue reagent. Following incubation (2 h at 37 °C), the fluorescent product was measured (excitation: 540 nm, emission: 586 nm) using Cary Eclipse spectrophotometer (Agilent). In each experiment, the fluorescence values were normalized to those resulting from 6 × 10^4^ cells.

### Metabolic parameter calculations

#### Cell respiration parameters

We applied the equations reported in Table 1 to calculate the most informative respiration parameters (non-mitochondrial respiration, basal and maximal mitochondrial respiration, spare respiratory capacity, i.e. the ability of a cell to meet an increased energy demand) derived from changes in OCR profile in response to the injection of the metabolic modulators (FCCP and rotenone/antimycin A), as previously described [27].

**Table 1:** Equations used to calculate the cell respiration parameters

Parameter	Equation
Non-mitochondrial respiration	Minimum OCR value after Rotenone/Antimycin A injection
Basal respiration	(Last measured OCR value before first injection)–(Non-mitochondrial respiration)
Maximal respiration	(Maximum measured OCR value after FCCP injection)–(Non-mitochondrial respiration)
Spare respiratory capacity	(Maximal respiration)–(Basal respiration)

#### Proton production rate (PPR) parameter

To enable conversion of the acidification rate (ECAR; mpH/min) to PPR (pmol H^+^/min) values, we measured the buffering power of the assay medium by performing a titration with HCl (Seahorse XFp Analyzer, Agilent), as previously described [20]. Briefly, the assay consisted of four sequential HCl (0.2 mM) injection steps (Seahorse XFp Analyzer) which led to a progressive pH decline. The pH variations were recorded and differences in pH (ΔpH) were calculated after each HCl injection. Since pH responded pseudo-linearly to H^+^, a regression line between ΔpH and H^+^ nmol of the measuring volume (2.28 µL) was calculated. The slope of the regression line is the buffering factor (BF) of the medium.

ECAR values (mpH/min) were converted to PPRs (pmol H^+^/min), by applying the Eq. 1, as reported by Mookerjee SA and collaborators [20]:

1 $$PPR = ECAR{ }\left( {\frac{{{\text{mpH}}}}{{{\text{min}}}}} \right) \div BF\left( {\frac{mpH}{{pmol{ }H + }}} \right)$$

where ECAR = extracellular acidification rate (mpH/min), BF = buffering factor (mpH/pmol H^+^).

Since live cells present two main sources of PPR (anaerobic glycolysis and CO_2_, which is hydrated to H_2_CO_3_ that dissociates in HCO_3_^−^ + H^+^) [20], the PPR values can be expressed with Eq. 2:

2 $$PPR_{tot} = PPR_{glyc} + PPR_{resp}$$

where the total PPR (PPR_tot_) equals the sum of anaerobic glycolytic PPR (PPR_glyc_) and respiratory PPR (PPR_resp_).

Therefore:

3 $$PPR_{glyc} = PPR_{tot} - PPR_{resp}$$

where PPR_resp_ was defined by Mookerjee SA [20] and collaborators in Eq. 4:

4 $$PPR_{resp} = (10^{pH - pK1 } /\left( {1 + 10^{pH - pK1 } } \right))\left( {maxH^{ + } /O_{2} } \right)\left( {OCR_{tot - } OCR_{rot/antA} } \right)$$

where OCR_tot_ = oxygen consumption rate measured by the instrument at each point (pmol O_2_/min); OCR_rot/antA_ = non-mitochondrial respiration; Max H^+^/O_2_ = the maximum H^+^ released in the medium per O_2_ consumed (and CO_2_ generated) by respiration (resumed in Table 2); pK1 = the dissociation equilibrium constant of CO_2_ to HCO_3_^−^ + H^+^, which is equal to 6.093 at 37 °C.

**Table 2:** Reactions of substrates complete oxidation

Metabolic substrates in the culture medium	Reaction of oxidation	Max H^+^/O_2_
Glucose (5.5 mM)	C_6_H_12_O_6_ + **6O**_**2**_ → 6CO_2_ + 6H_2_O 6CO_2_ + 6H_2_O → 6HC ${\text{O}}_{3}^{ - }$ + **6** ${\mathbf{H}}^{ + }$	1.00
Glutamine (5.5 mM)	C_5_H_10_N_2_O_3_ + **4,5O**_**2**_ → 5CO2 + 2H_2_O + 2NH_3_ 5CO_2_ + 2NH_3_ + 5H_2_O → 5HC ${\text{O}}_{3}^{ - }$ + 2 N ${\text{H}}_{4}^{ + }$ + **3** ${\mathbf{H}}^{ + }$	0.67
Pyruvate (0.37 mM)	C_3_H_3_ ${\text{O}}_{3}^{ - }$ + ${\mathbf{H}}^{ + }$ + **2,5O**_**2**_ → 3CO_2_ + 2H_2_O 3CO_2_ + 3H_2_O → 3HC ${\text{O}}_{3}^{ - }$ + **3** ${\mathbf{H}}^{ + }$	0.80
Stearic Acid (100 µM)	C_18_H_35_ ${\text{O}}_{2}^{ - }$ + ${\mathbf{H}}^{ + }$** + 26O**_**2**_ → 18CO_2_ + 18H_2_O 18CO_2_ + 18H_2_O → 18HC ${\text{O}}_{3}^{ - }$ + **18** ${\mathbf{H}}^{ + }$	0.65

As indicated in [20] we assumed that the oxidation of the two substrates mainly represented in the seahorse medium (glucose and glutamine both 5.5 mM), equally contribute to Krebs cycle. As thoroughly explained and derived in [20] therefore, the weighted max H^+^/O_2_ value of the complete seahorse medium is bound to be 0.90.

The low concentrations of pyruvate (0.37 mM) and stearic acid (0.1 mM) do not substantially alter this value.

#### ATP production rate

ATP production rate was computed starting from extracellular measurements of rates of acidification (ECAR) and oxygen consumption, under the assumption that glucose and glutamine were the only two quantitatively significant substrates undergoing oxidation, as described by Mookerjee SA and collaborators [21]. Total ATP production was defined as the sum of ATP from anaerobic glycolysis (ATP_glyc_) and ATP derived from mitochondrial oxidative phosphorylation (ATP_oxphos_). The flux of ATP derived from anaerobic glycolysis was assumed to be one and the same with the PPR_glyc_, as easily derived from the stoichiometry of anaerobic glycolysis by Mookerjee et al*.* [21].

#### *ATP*_*oxphos*_* calculation*

ATP_oxphos_ is defined as the ATP produced by oxidative phosphorylation and, in our experimental setting, is the sum of glucose-driven ATP (ATP_glc_) and glutamine-driven ATP (ATP_gln_). Therefore: $ATP_{oxphos} = ATP_{glc} + ATP_{gln}$.

As indicated in [21], the two substrates were assumed to contribute to the TCA flux with the molar ratio glucose/glutamine of 2.5, which leads to the following equation, previously reported in [21]:

5 $$ATP_{glc} = OCR_{mito} { } \times 0.714 \times \frac{P}{O}{ }glc \times 2$$

6 $$ATP_{gln} = OCR_{mito} { } \times 0.286 \times \frac{P}{O}{ }gln \times 2$$

In which:

- OCR_mito_ is the OCR measured by the instrument (at each step) subtracted the OCR_rot/antA;_
- 0.714 and 0.286 are the proportions of glucose and glutamine, respectively, oxidized, in line with the assumption that the molar ratio of the contribution of glucose and glutamine to the TCA flux is 2.5;
- P/O_glc_ and P/O_gln_ are the P/O ratios (i.e. the maximal number of ADP molecules converted to ATP per each atom of oxygen consumed in the ETC) of glucose and glutamine, respectively [21].

### Bioenergetic plots

By plotting the PPR_glyc_ (x axis) and OCR values (y axis) as coordinates in a scatter plot we represent the (in)capacity of a cell population to switch from an oxidative to a glycolytic metabolism (and vice versa)*.* The higher the intercept of the line generated by the experimental points, the greater is the maximal capacity of the cell to generate ATP via the oxidative phosphorylation. The steeper the slope of the same line, the less is the capacity of the cell to switch to anaerobic glycolysis to generate ATP, i.e. to function under severe hypoxic conditions. We called this graph the *respiration-glycolysis switch plot*.

A reduction of the intercept value on the y axis generally can be interpreted as a limitation of cell capacity to generate ATP (and vice versa), given the much higher efficiency of oxidative phosphorylation than anaerobic glycolysis in producing ATP. A reduction in the intercept of the x axis can be interpreted as a limitation of the maximal flux of anaerobic glycolysis (and vice versa).

Similarly, in order to represent the relative contribution of mitochondrial respiration and anaerobic glycolysis in supplying ATP for cellular needs, we plotted ATP_glyc_ (x axis) and ATP_oxphos_ (y axis) values in a scatter plot [21], thereby generating the *ATP source plot*. The graph bisector collects all the points at which ATP production derives 50% from glycolysis and 50% from oxidative metabolism. The absolute positions of points in the graph characterize the cell bioenergetic phenotype: points below the bisector line represent cells deriving ATP preferentially from glycolysis, conversely, points above the bisector represent cells deriving more than 50% of ATP from oxidative metabolism.

### Statistical analysis

All data are presented as mean ± SEM. Differences among groups were identified using repeated measure ANOVA (followed by Dunnet post-hoc). Statistical significance was set at p < 0.05 (two-sided). Data analysis was performed using SPSS version 24.0 (SPSS Inc/IBM, Chicago, Ill, USA) and GraphPad Prism version 5 (GraphPad Software Inc.).

## Results

### Effects of stearic acid on bioenergetic metabolism

We previously demonstrated that physiologic concentrations of SA induced inflammation, oxidative and ER stress and functional alterations in MAC [8]. Here, we explored, in the same experimental setting, whether SA-induced effects are accompanied by MAC bioenergetic phenotype alterations.

**Fig. 1:**
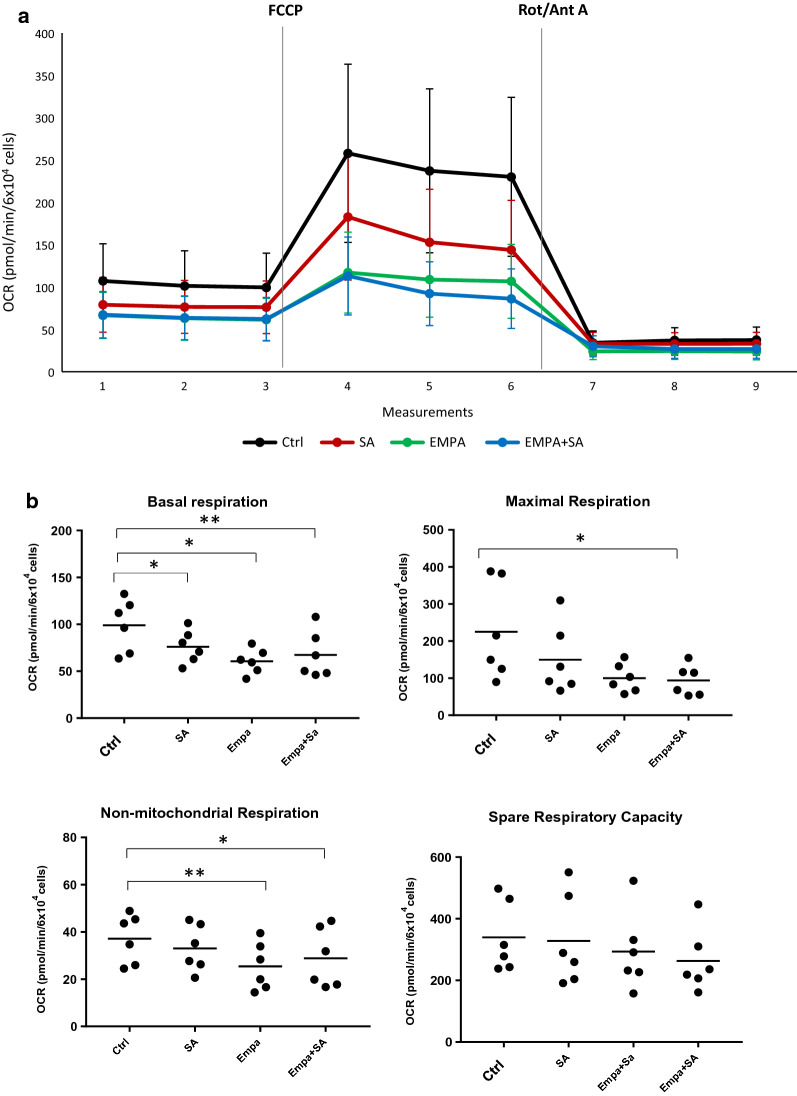
Mito Stress Test and respiration parameters. Mito Stress Test was assessed in MAC pre-treated with empagliflozin (EMPA) 100 µM for 1 h followed by 3 h of 100 µM SA incubation (**a**); cell respiration parameters were calculated (**b**), following the equations reported in Table 1. Metabolic stressors (2 μM FCCP; 1 μM Rot/Ant A) were sequentially injected. All OCR data were normalized to 6 × 10^4^ cells and expressed as mean ± SEM from 6 independent experiments (*p < 0.05 vs Ctrl) (**p < 0.01 vs Ctrl). Ctrl = control culture condition; SA = stearate; Empa = empagliflozin FCCP = carbonyl cyanide 4-(trifluoromethoxy)phenylhydrazone; Rot/Ant A = rotenone + antimycin A

The Mito Stress Cell Test showed that SA diminished OCR profile, especially at baseline and after FCCP injection, compared to control (Fig. 1A). Specifically, by calculating respiration parameters (with the equations reported in Table 1), we documented that SA induced a reduction in basal respiration compared to control (p < 0.05; Fig. 1B). No differences were observed in the other OCR-derived respiration parameters.

**Fig. 2:**
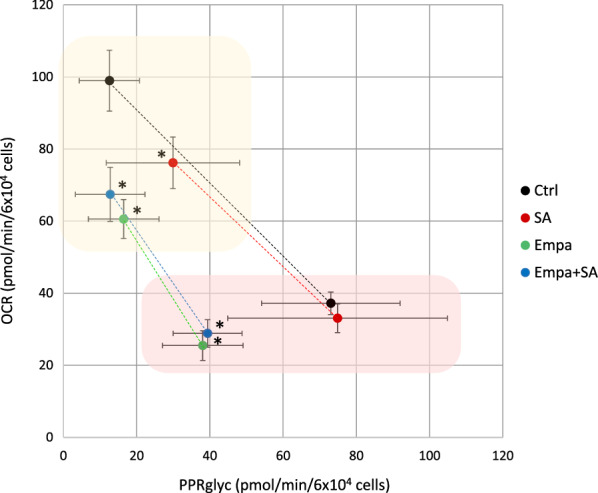
Effects of SA and EMPA on respiration-glycolysis switch plot. The graph shows OCR (y axis) and PPR_glyc_ (x axis) values of MAC pre-treated with EMPA 100 µM for 1 h followed by 3 h of 100 µM SA incubation. Yellow and red areas represent MAC at baseline and after complete ETC inhibition, respectively. All OCR and PPR_glyc_ data were normalized to 6 × 10^4^ cells and expressed as mean ± SEM from 6 independent experiments (*p < 0.05 vs Control).Ctrl = control culture condition; Empa = empagliflozin, SA = stearate

To assess the effect of SA on MAC bioenergetic flexibility, we used the *respiration-glycolysis switch plot* (Fig. 2), in which we represent MAC culture conditions at baseline (yellow area) and following complete ETC inhibition (red area). SA (red dots) induced a reduction in OCR at baseline compared to control (− 23% ± 9% vs control; p < 0.05). No differences were observed in OCR after ETC inhibition and in PPR_glyc_ values.

**Fig. 3:**
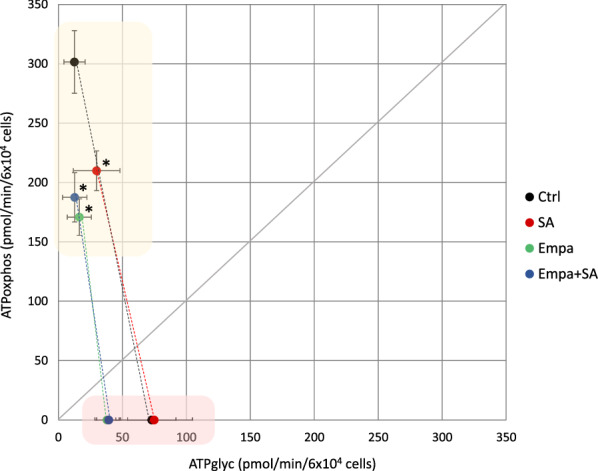
Effects of SA and EMPA on ATP source plot. The graph shows ATP_oxphos_ (y axis) and ATP_glyc_ (x axis) values of MAC pre-treated with EMPA 100 µM for 1 h followed by 3 h of 100 µM SA incubation. Yellow and red areas represent MAC at baseline and after complete ETC inhibition, respectively. All data were normalized to 6 × 10^4^ cells and expressed as mean ± SEM from 6 independent experiments (*p < 0.05 vs Control). Ctrl = control culture condition; Empa = empagliflozin, SA = stearate

The *ATP source plot* was drawn to assess the effects of SA on cell bioenergetics (Fig. 3). It showed that a) MAC mainly rely on oxidative metabolism for ATP production at baseline and on anaerobic glycolysis—by experimental design—following ETC inhibition; and b) SA (red dots) caused a reduction in ATP_oxphos_ values (− 30% ± 8% vs control; p < 0.05) at baseline, compared to control (black dot). No differences were found in ATP_glyc_ values, in line with the aforementioned PPR_glyc_ results.

### Effects of empagliflozin on bioenergetic metabolism

As we recently showed that empagliflozin curbs SA-induced lipotoxicity in MAC [19], we herein investigated whether EMPA could induce concomitant changes in MAC metabolism. Surprisingly, Mito Stress Cell Tests revealed that EMPA-treated cells—both in presence/absence of SA—induced a massive reduction in the OCR trajectory, both at baseline and after FCCP injection (Fig. 1A).

The Mito stress-derived respiration parameters confirmed that EMPA—both in presence/absence of SA—caused a reduction of non-mitochondrial respiration rate (p < 0.05 and p < 0.01, respectively) and a strong diminution of basal respiration rate (p < 0.01 and p < 0.05, respectively) compared to control. Furthermore, EMPA plus SA reduced maximal respiration rate (p < 0.05) *vs* control (Fig. 1B).

Consistently, the *respiration-glycolysis switch plot* showed that EMPA induced a noticeable OCR reduction—both at baseline (-39% ± 9% EMPA vs control; − 32% ± 11% EMPA + SA *vs* control; both p < 0.05) and following ETC inhibition (− 32% ± 16% EMPA *vs* control; − 22% ± 13% EMPA + SA vs control; both p < 0.05) (Fig. 2).

It should be noticed that the SA-induced OCR reduction was accompanied by an appropriate increase in glycolysis (Fig. 2), thereby keeping cells over the physiological line of respiration-glycolysis switch (the points of SA-treated cells fell on the same line as the control cells). On the contrary, the points of the EMPA-treated MAC (green dots) fell on an entirely different line than control cells, a line characterized by a reduction in maximal OCR and an apparent, albeit not statistically significant, reduction in PPR_glyc_.

Similarly, the *ATP source plot*, used to represent EMPA effects on MAC bioenergetics (Fig. 3), showed that EMPA was associated to lower ATP_oxphos_ values (− 43% ± 9% EMPA vs control; − 38% ± 11% EMPA + SA vs control; p < 0.05) at baseline. The numerical reduction in ATP_glyc_ production was not statistically significant.

### Effects of amiloride on bioenergetic metabolism

We recently [19] pointed to NHE inhibition as the mechanism responsible for the effects of SGLT-inhibitors in curbing SA-induced inflammation and oxidative stress in MAC. To investigate the potential role of NHE inhibition also in the metabolic action of EMPA, we assessed the effects of amiloride—the NHE inhibitor mimicking the anti-inflammatory action of EMPA—in the presence/absence of SA, on MAC bioenergetics.

**Fig. 4:**
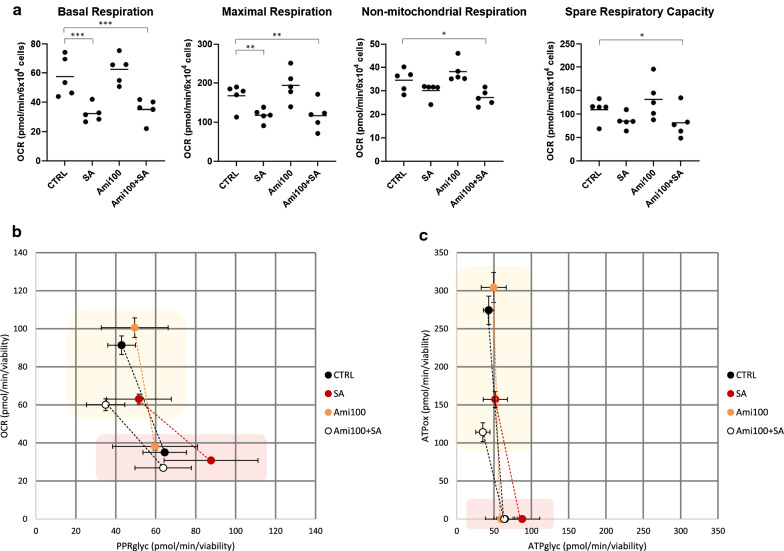
Effects of amiloride on MAC metabolism. The Mito Stress respiration parameters (**a**), the respiration-glycolysis switch plot (**b**), and the ATP source plot (**c**) of MAC pre-treated with amiloride 100 µM for 1 h followed by incubation with SA (100 µM) for 3 h are shown. Yellow and red areas represent MAC at baseline and after complete ETC inhibition, respectively. All data were normalized to 6 × 10^4^ cells and expressed as mean ± SEM from 6 independent experiments (* p < 0.05, ** p < 0.01, *** p < 0.005 vs Control). Ctrl = control culture condition; SA = stearate; Ami = amiloride

The Mito Stress Cell Tests showed that amiloride did not affect OCR values, neither at baseline, nor following FCCP injection in MAC. Accordingly, amiloride had a neutral effect on respiration parameters which remained unchanged compared to control and SA (Fig. 4A).

Consistently, the *respiration-glycolysis switch plot* and the *ATP source plot* showed no effects of amiloride both at baseline and following ETC inhibition (Fig. 4B and C) *vs* control and SA. These data suggest that NHE is not involved in the regulation of metabolic effects of EMPA.

## Discussion

In this study we tested the hypotheses that (1) SA-induced lipotoxic effects might alter also MAC metabolism and (2) SGLT2-I ameliorating effects of MAC lipotoxicity might be concomitant with coherent changes in cell bioenergetics.

Our data show that SA-induced lipotoxic damage (i.e. inflammation and oxidative stress) is accompanied by an impairment in mitochondrial-derived metabolism and energetics.

However, the compensatory capacity of MAC of this bioenergetic defect via anaerobic glycolysis stays the same. Unexpectedly, empagliflozin, which curbs lipotoxicity in MAC [19], induced even greater alterations in basal and maximal cellular respiration (and mitochondrial ATP production) than SA. Of note, while the anti-inflammatory effects of EMPA were supposed to be mediated by NHE inhibition [19], those regarding bioenergetics were NHE-independent.

To the best of our knowledge, this is the first study investigating MAC bioenergetic phenotype, which was shown to be highly oxidative at baseline. This is in line with macrophage [28], dendritic cell [29] and T lymphocyte [30] metabolic profiles, which mainly rely on mitochondrial respiration rather than on anaerobic glycolysis. The increase in glycolysis following ETC blocking—achieved with the addition of rotenone and antimycin A—was expected, as it is known that, in condition of hypoxia or mitochondrial stress, glycolysis is the only metabolic pathway able to rapidly provide ATP [31].

Lipotoxicity, primarily triggered by higher levels of saturated fatty acids, *in primis* palmitic acid (PA 16:0) and SA (18:0), is recognized as one of the main mechanisms at the base of the relation among metabolic disorders, endothelial dysfunction and increased CV risk. We previously showed that physiological (100 µM) concentrations of SA—but not of PA which was effective at 1 mM doses—caused inflammation, oxidative stress and apoptosis through endoplasmic reticulum stress and c-Jun N-terminal kinase activation in MAC [8].

In the present study, SA induces bioenergetic alterations in conditions—times and concentrations—in which it also triggers the aforementioned lipotoxic damages in MAC [8].

Lipotoxicity-associated metabolic alterations have been described in the literature; in particular PA is responsible for a reduction in the number and function of mitochondria together with a significant release of ROS and an impairment of ATP production due to a decrease in ETC complex II and IV activity in a macrophage cell line [32]. Similarly, the chronic exposure of human endothelial cells to physiological concentrations of PA induces, in addition to an increase in the inflammatory response and in the production of ROS, a decrease in mitochondrial membrane potential, dysfunction of the mitochondrial oxidative phosphorylation system with reduced ATP synthesis [33]. Further studies are warranted to elucidate whether SA and PA share the same mechanism underlying their metabolic action.

It is worth of notice that, in the present study, the SA-induced reduction in mitochondrial respiration was supplied by an apparently appropriate compensatory increase in glycolysis. This metabolic feature is named *Pasteur effect,* defined as the glycolytic activation following the reduction/inhibition of mitochondrial respiration. Our data suggest that physiological concentration of SA may trigger metabolic changes, but with no evident gross damage to the general bioenergetic balance of MAC.

However, the reduction in oxidative metabolism brought about by SA might still induce a lipotoxic damage in the mitochondrion, via saturated fatty acid induced opening of the permeability transition pore, which is a high conductance mitochondrial channel and in turn causes the release in the cytosol of apoptogenic proteins (e.g. cytochrome c) [34, 35].

Our data add bioenergetic changes to the array of potential harmful effects exerted by stearic acid at physiologic concentrations in MAC, highlighting the association between metabolic modification and MAC *secretome*/function. In light of the pivotal role played by MACs in the maintenance of endothelial homeostasis, these results might turn out to be preliminary to future studies aiming at identifying new metabolic targets for innovative therapeutic strategies for the treatment of lipotoxicity-associated conditions (e.g. insulin resistance, obesity and T2D) and CV diseases. Our evidence regarding the protective role of EMPA in blunting SA-induced lipotoxicity (inflammation and oxidative stress) in MAC [19], is supported by a recent study in rodent renal proximal tubule cells, in which EMPA also ameliorates PA-induced lipotoxicity, with regard to cell viability and inflammation, by inhibiting a PPAR-γ/CD36 pathway [37]. The most intriguing finding of the present study is that the reduction of the lipotoxic damage by EMPA is accompanied by a strong fall in mitochondrial respiration and ATP production, without significantly affecting the glycolytic pathway in MAC. Therefore, EMPA does not reverse, and it might even worsen, SA-induced fall in mitochondrial O_2_ consumption and ATP production, proving that the protective effect of EMPA against SA-induced lipotoxicity is unlikely to be mediated through bioenergetic metabolism. Indeed, empagliflozin deeply affects OCR and ATP_oxphos_, thereby documenting a severe defect of mitochondrial bioenergetic metabolism. EMPA depressive effect on cell respiration is in accordance with prior metabolic studies performed with other SGLT2-I [17, 18, 36]. Canagliflozin mediated dual inhibition of mitochondrial glutamate dehydrogenase and ETC complex I in human renal proximal tubule epithelial cell [17] and at a concentration of 30 μM impaired oxygen consumption and ATP flux in mouse hepatocyte and in human embryonic kidney cell line [36]. Furthermore, the treatment with dapagliflozin reduced TCA cycle and ATP production in isolated mitochondria from muscle biopsy of T2D patients, compared to placebo [18]. These evidences suggest that SGLT2-I, as a class effect, may strongly inhibit mitochondrial respiration and ATP turnover. In rodents SGLT2-I can bind to Na^+^/H^+^ exchangers (NHE) [38] and, by inhibiting myocardial NHE flux, improve cardiac pump function. In line with these findings, we also proposed NHE as the molecular transducer(s) of SGLT2-I-mediated anti-inflammatory and anti-oxidative actions in lipotoxic MAC [19]. The SGLT2-I-induced fall in oxygen consumption reported in the present study could be, at least partially, ascribed to a putative beneficial effect, i.e. the impaired production of ROS. These substances account for a part of the oxygen consumed by the mitochondrion. Several studies ascribe this property to both SGLT2-I [15, 39] and NHE inhibitors [40, 41].

Nevertheless, we found that NHE inhibition by amiloride did not affect MAC bioenergetic parameters and did not reverse SA-induced metabolic alterations, suggesting that the (partial) block of NHE mediates the anti-inflammatory—but not the metabolic—effects of EMPA in SA-treated MAC. Since also EMPA does not reverse SA-induced metabolic alterations, it is unlikely that in human MAC it acts through inhibition of PPARγ/CD36, because this mechanism would prevent SA entry into and metabolism by the cell. Further experiments are needed to unravel the specific molecular mechanism underlying the metabolic changes induced by EMPA in MAC.

We recognize that our study has several limitations: (1) EMPA at concentrations (100 µM) higher than the pharmacologic levels reported to be attained in humans (1 µM); nevertheless, intra-cellular concentrations of SGLT2-I are unknown and > 10 µM concentrations of SGLT2-I have been used in previous mechanistic studies [36, 42]; (2) The causal relationship between SA-induced bioenergetic changes and alterations in inflammation/function of MAC was not tested in the present study and needs to be proven (3) further targeted experiments are needed to unveil the exact significance and mechanism(s) of empagliflozin action on cell bioenergetics.

## Conclusions

In conclusion, our study shows that, in human MAC, stearic acid induces alterations in MAC bioenergetics, concomitantly with inflammation and oxidant stress. Furthermore EMPA, at concentrations known to counteract stearic acid induced lipotoxicity, inhibits mitochondrial respiration and ATP turnover in a NHE-independent manner, thereby suggesting that the protective effect of EMPA against SA-induced lipotoxicity is unlikely to be mediated through the changes in bioenergetic metabolism.

## Data Availability

The datasets used and/or analyzed during the current study are available from the corresponding author on reasonable request.

## Abbreviations

AMPK: AMP-activated protein kinase
ATP_glc_: Glucose-driven ATP
ATP_gln_: Glutamine-driven ATP
ATP_glyc_: ATP derived from anaerobic glycolysis
ATP_oxphos_: ATP derived from mitochondrial oxidative phosphorylation
BF: Buffering factor
CV: Cardiovascular
ECAR: Extracellular acidification rate
EGM-2: Endothelial cell growth medium-2
ETC: Electron transport chain
MAC: Myeloid angiogenic cells
MNCs: Mononuclear cells
NHE: Sodium-proton exchangers
Nlrp3: NOD-like receptor-3
OCR: Oxygen consumption rate
OCR_rot/antA_: Non-mitochondrial respiration
OCR_tot_: Total oxygen consumption rate
P/O_glc_: P/O ratio of glucose
P/O_gln_: P/O ratio of glutamine
PA: Palmitic acid
PPR: Proton production rate
PPR_glyc_: Glycolytic PPR
PPR_resp_: Respiratory PPR
ROS: Reactive oxygen species
SA: Stearic acid
SGLT2-I: Sodium-glucose cotransporter-2 inhibitors
T2D: Type 2 diabetes mellitus
